# miR-205 Regulates Tamoxifen Resistance by Targeting Estrogen Receptor Coactivator MED1 in Human Breast Cancer

**DOI:** 10.3390/cancers16233992

**Published:** 2024-11-28

**Authors:** Bin Ouyang, Mingjun Bi, Mahendra Jadhao, Gregory Bick, Xiaoting Zhang

**Affiliations:** 1 Department of Cancer Biology, University of Cincinnati College of Medicine, Cincinnati, OH 45267, USA; ouyangb@ucmail.uc.edu (B.O.); bimn@ucmail.uc.edu (M.B.); or jadhaomg@ucmail.uc.edu (M.J.); bickgp@ucmail.uc.edu (G.B.); 2 Breast Cancer Research Program, University of Cincinnati Cancer Center, Cincinnati, OH 45267, USA

**Keywords:** breast cancer, MED1, ERα, HER3, miR205, drug resistance, tamoxifen

## Abstract

**Background/Objectives**: Estrogen receptor-α coactivator MED1 is overexpressed in 40–60% of human breast cancers, and its high expression correlates with poor disease-free survival of patients undergoing anti-estrogen therapy. However, the molecular mechanism underlying MED1 upregulation and activation in breast cancer treatment resistance remains elusive. **Methods**: miRNA and mRNA expression analysis was performed using the NCBI GEO database. MED1 targeting and its impact on therapy resistance was evaluated in control and tamoxifen-resistant breast cancer cell lines by miR-205 overexpression and inhibition. Immunoblotting, chromatin immunoprecipitation, and luciferase reporter assays were used to understand the molecular mechanism of MED1-mediated tamoxifen resistance. Mice xenograft models were used to validate treatment efficacy and molecular mechanisms in vivo. **Results**: miR-205 was found to directly target and suppress the expression of MED1 through bioinformatic analyses and experimental validations. An inverse correlation of miR-205 and MED1 was observed in breast cancer patients with high MED1/low miR-205, indicative of poor prognosis in long-term anti-estrogen treatment. Furthermore, the depletion of miR-205 was observed in tamoxifen-resistant breast cancer cells overexpressing MED1. The restoration of miR-205 expression attenuated MED1 expression and re-sensitized cells to tamoxifen both in vitro and in vivo. Interestingly, miR205 was also found to target another key regulatory gene, HER3, which drives PI3K/Akt signaling and MED1 activation by phosphorylation. Importantly, we found ER target gene transcription and promoter cofactor recruitment by tamoxifen can be reversed by induced miR205 expression. **Conclusions**: Altogether, miR-205 functions as a negative regulator of MED1 and HER3, affecting the regulation of the HER3-PI3K/Akt-MED1 axis in anti-estrogen resistance, and could serve as a potential therapeutic regime to overcome treatment resistance.

## Simple Summary

Estrogen receptor coactivator MED1 is found in abnormally high levels in 40–60% of breast cancers and is linked to worse outcomes for patients receiving anti-estrogen treatments. This study investigates how MED1 is overexpressed and overactive in breast cancer and contributes to treatment resistance. We observed that a small RNA, miR-205, is inversely correlated with MED1 expression levels in breast cancer patients, with low levels of miR-205 and high levels of MED1 associated with poor survival. We further found that tamoxifen-resistant human breast cancer cell lines have lower miR-205, leading to higher MED1 and resistance to treatment in vitro and in vivo. Restoring miR-205 levels in tamoxifen-resistant cancer cells reduced MED1 expression and enhanced tamoxifen sensitivity by disrupting MED1 expression and activation by the HER3-PI3K/Akt-MED1 axis. Overall, our studies support the miR-205 regulation of MED1 in anti-estrogen resistance and as promising treatment targets.

## 1. Introduction

Breast cancer is the most common malignancy in women and one of the leading causes of cancer deaths among U.S. women. Approximately two-thirds of primary breast cancers express estrogen receptor-α (ERα), which contributes to tumor growth and progression [1,2,3,4]. Therefore, the disruption of the ERα signaling network has become the main therapeutic strategy for these ERα-positive breast cancer patients [4,5]. The selective ERα modulators, including tamoxifen, can competitively inhibit the interaction of estrogen with ERα and thus repress ERα activity [6]. Over the past few decades, tamoxifen remains one of the most widely used endocrine therapies for ERα-positive cancers [4]. Although the use of tamoxifen significantly improves the survival of women with early-stage breast cancer [7], unfortunately, not all ERα-positive tumors respond to this therapy (de novo resistance), and almost all patients with metastatic ERα-positive disease eventually develop endocrine resistance (acquired resistance) [7,8,9]. Thus, understanding the molecular mechanism underlying the tamoxifen resistance and identifying new ways to overcome such resistance is of critical clinical importance.

Mediator Subunit 1 (MED1) is a key ERα coactivator and directly interacts with ERα via two signature LxxLL motifs [10,11,12,13]. Animal model studies indicated that MED1 plays a vital role in mediating estrogen functions during pubertal mammary gland development, luminal epithelial cell differentiation, and progenitor/stem cell determination [10,12,13]. MED1 has been reported to be overexpressed or amplified in a high percentage (40–60%) of human primary breast cancer [13,14]. Importantly, the overexpression of MED1 highly correlates with the poor survival of breast cancer patients undergoing tamoxifen treatment [15,16]. Our recent studies have further demonstrated that MED1 plays a critical role in tamoxifen resistance by direct crosstalk with the receptor tyrosine kinase HER2 signaling pathway in human breast cancer cells [11,17]. However, despite these findings, the molecular mechanism underlying both the upregulation and activation of MED1 in tamoxifen resistance still remains poorly defined.

MicroRNAs (miRNAs) are a class of short (~22 bp), single-stranded, non-coding RNAs, which primarily bind the 3′untranslated region (UTR) of mRNAs at sequences that have imperfect or perfect complementarity, leading to the post-transcriptional silencing or mRNA degradation of the respective genes [18,19]. The dysregulation of miRNAs is frequently associated with the initiation and progression of human breast cancer [20,21,22]. Recent miRNA expression profiling studies from a large number of human breast tumors have uncovered the oncogenic and tumor-suppressive roles of differential miRNAs [21,22]. There is also increasing evidence supporting a role for miRNAs in mediating anti-estrogen resistance of human breast cancer [23,24,25,26]. It has been reported that the overexpression of miR-221/222, miR-101, and miR-519a were able to confer tamoxifen resistance [27,28,29], while the re-expression of miR-342, let-7, miR-375, miR-873, or miR-320a can induce the tamoxifen sensitivity of otherwise resistant breast cancer cells [30,31,32,33,34].

Here, we present evidence that miR-205 can directly regulate the expression of the ER transcriptional coactivator MED1 to confer tamoxifen sensitivity. Importantly, the expression of miR-205 inversely correlates with MED1 in the breast cancer cell lines and human breast cancer patient samples. Further, we show that miR-205 can also regulate the phosphorylation/activation of MED1 through downregulating the HER3/PI3K/Akt pathway, thus inhibiting the recruitment of ER cofactors to the target gene promoters and regulating tamoxifen treatment response of breast cancer cells. Significantly, the re-expression of miR-205 in vivo in orthotopic xenograft mouse models represses the expression of MED1 and confers sensitivity to otherwise tamoxifen-resistant breast cancer cells. Together, this study provides novel insights into miR-205 and its interplay with the HER3-PI3K/Akt-MED1 axis in mediating tamoxifen resistance and the potential to target this regulatory pathway for future therapeutic applications.

## 2. Materials and Methods

### 2.1. Cell Culture

The human breast cancer cell lines MCF-7 and BT474 were purchased from the American Type Culture Collection (ATCC, Manassas, VA, USA). The 293T cell line was obtained from Invitrogen (Carlsbad, CA, USA). MCF-7, BT474, and 293T cells were maintained in Dulbecco’s Modified Eagle’s Media (DMEM) medium (Hyclone, Logan, UT, USA) supplemented with 10% fetal bovine serum (FBS) and 1% pen/strep at 37 °C with 5% CO_2_ in tissue culture incubators. MCF7, BT474, and 293T cells were multiplied/frozen and used at low passage numbers not exceeding 15 passages. The 4-Hydroxytamoxifen (4-OHT)-resistant MCF7 cell line (MCF-7/TAM) was a kind gift from Dr. Kenneth P. Nephew (Indiana University). The MCF7/TAM cells were generated as described previously; briefly, MCF7 cells were maintained in a hormone-free culture medium supplemented with 10^−7^ mol/L 4-OHT for 12 months [35,36]. MCF7/TAM cells were used in working culture conditions, so as not to exceed 5 passages for experiments. PI3K inhibitor LY294002 and Doxycycline were purchased from LC laboratories and Fisher Scientific, respectively. 17β-estradiol (E2) and 4-OHT were purchased from Sigma (Sigma-Aldrich, St. Louis, MO, USA). For experiments involving E2 or 4-OHT treatments, cells were cultured in phenol red-free DMEM supplemented with 10% charcoal-stripped FBS for 3 days before the treatments.

### 2.2. Plasmids and Lentiviral Vector Construction

The plasmids pcDNA3.1-MED1, pLKO.1-MED1 short hairpin RNA (shRNA), and pLKO.1-scramble shRNA were described previously [11]. The plasmid pLKO.1-HER3 shRNA was purchased from Cincinnati Children’s Robotic Lenti-Library Core. To construct miR-205 lentiviral expression plasmids, the *miR-205* gene was amplified from 293T genomic DNA by the high-fidelity polymerase Phusion enzyme (Invitrogen). The PCR products were then cloned into lentiviral vector pCDH-CMV-MCS-EF1-Puro (System Biosciences, Palo Alto, CA, USA). High-titer lentiviruses were generated by the transient transfection of 293T cells using pCDH-CMV or pLKO.1 constructs with psPAX2 and pMD2.G plasmids according to the manufacturer’s instructions (System Biosciences). The miR-205 inhibitor and control oligonucleotides were purchased from GeneCopoeia^TM^. The pLKO-Tet-on-miR-205 plasmids were constructed by inserting the miR-205 gene into pLKO-Tet-on vectors. The miR-205 putative binding sites and desired 3′UTR mutations of MED1 and HER3 were amplified by PCR from human 293T cells and then were cloned into pMIR-REPORT™ Luciferase miRNA Expression Reporter Vector. All inserts were confirmed by DNA sequencing (Genewiz, South Plainfield, NJ, USA).

### 2.3. Dual-Luciferase Reporter Assay

For transfection, six thousand 293T cells were seeded in triplicate in 96-well plates. After overnight incubation, cells were transfected with 20 ng of pMIR-REPORT bearing the wild type and mutant of 3′UTR of the target gene, and 20 ng of pCDH-miR-205, miR-205 inhibitor, or scrambled control using Lipofectamine^®^ 2000 Transfection Reagent (Invitrogen) according to the manufacturer’s protocol. The pRL-CMV plasmid (1 ng) was co-transfected to serve as a transfection efficiency control. Luciferase activity was measured using a dual luciferase reporter assay system (Promega, Madison, WI, USA) 48 h post-transfection. The results were confirmed with three independent experiments, and statistical analysis was performed by pairwise Student’s *t*-test, with *p* < 0.05 considered significant.

### 2.4. RNA Isolation and Real-Time PCR

Total RNAs were extracted from cultured cells and tissue samples using the RNeasy Mini kit (Qiagen, Hilden, Germany) or TRIzol reagent (Invitrogen) according to the manufacturer’s manual. A 1 µg amount of total RNA was reverse transcribed to cDNA using SuperScript^®^ III Reverse Transcriptase (Invitrogen). For miRNA, miRNeasy Mini Kits (Qiagen) were used, and isolated RNAs were converted to cDNA with the MystiCq^®^ MicroRNA^®^ Quantitation System (Sigma-Aldrich) according to the manufacturer’s manual. Real-time PCR was performed using SYBR Green PCR Master Mix on an Applied Biosystems 7900 Sequence Detection System. Relative gene expression was analyzed using the 2^−ΔΔCT^ method by normalization to the endogenous control. GAPDH was used as an internal control for the detection of mRNA expression level, while U6 small nuclear RNA was used as an endogenous control for miRNA expression analysis. Primer sequences are provided in Supplement Table S1.

### 2.5. Immunoblotting

Cell lysates were prepared by incubating cells in RIPA lysis buffer containing protease inhibitors (Roche, Basel, Switzerland), 0.1M DTT, 0.1M PMSF, and phosphatase inhibitors (Sigma) for 30 min on ice. Lysates were cleared by centrifugation at 14,000 rpm (10 min) and the supernatant was collected. A total of 30 μg of cell extracts, as measured by standard Bradford assay, were separated by SDS-PAGE, electrotransferred on PVDF membranes, and subjected to Western blot analyses with the following primary antibodies: anti-Akt (1:1000), anti-p-Akt (1:500; Ser473), and anti-HER3 (1:1000) antibodies from Cell Signaling, while the anti-MED1 (1:1000) ^13^, anti-p-MED1 (1:1000) [37], and anti-β-actin (1:8000) antibodies were used as control, purchased from Sigma. Host-specific HRP-conjugated secondary antibodies were purchased from Santa Cruz Biotechnology (Dallas, TX, USA). Images were developed with Pierce™ ECL Western blotting Substrate (Thermo Scientific, Waltham, MA, USA) and exposed to X-ray film.

### 2.6. Chromatin Immunoprecipitation (ChIP)

ChIP analysis was performed as described previously [38]. In brief, 6 × 10^6^ cells were treated with 1% formaldehyde for 10 min at room temperature to crosslink DNA and proteins. The reaction was terminated by adding a stop buffer and incubated at room temperature for 5 min. The lysates were incubated for 20 min on ice and sonicated to generate an average DNA size of 0.5–1 kb. The samples were centrifuged at 14,000 rpm at 4 °C for 10 min and the supernatant was collected. With pre-cleared samples, immunoprecipitation was performed with Protein A/G PLUS-Agarose (Santa Cruz) and anti-MED1, anti-p-MED1, anti-N-CoR (Santa Cruz), anti-SMRT (Santa Cruz), RNA polymerase II antibodies, or normal mouse IgG and rabbit IgG (as a negative control). Protein–DNA crosslinks were reversed, and DNA was recovered by phenol/chloroform/isoamyl-alcohol purification and used for real-time qPCR. The primers for pS2, cyclin D1, and c-myc promoter are listed in Supplement Table S1.

### 2.7. Cell Proliferation and Clonogenic Assay

Cell proliferation was assessed by MTT (3-[4, 5-dimethyl-2-thiazolyl]-2, 5-diphenyl-2H-tetrazolium bromide, Sigma) assay as described previously, with minor modifications [38]. 2000 cells/well were seeded in a 96-well plate and treated with a vehicle, E2 or 4-OHT, as indicated. MTT was added to the medium to a final concentration of 0.5 mg/mL and incubated for 4 h at 37 °C. The medium was then removed and 0.2 mL DMSO was added. After incubation for another 30 min at room temperature, the absorbance was measured at 570 nm using a Synergy II spectrophotometer (Biotek, Winooski, VT, USA). To determine clonogenic ability, cells were allowed to grow for 2 weeks to form colonies. The colonies were then fixed with 10% formaldehyde, stained with 1% crystal violet (Sigma), and counted.

### 2.8. Immunohistochemistry

Immunohistochemistry (IHC) staining was carried out essentially as previously described [11]. Briefly, the slide was first deparaffinized and subjected to heat-induced antigen retrieval using citrate buffer (pH 6.0). After blocking with 5% goat serum, the sections were then incubated with primary antibodies overnight at 4 °C, followed by extensive washes. The slide was subsequently treated with biotinylated secondary antibodies and then developed using streptavidin-conjugated horseradish peroxidase with 3,3′-diaminobenzidine (DAB) as the substrate (VECTASTAIN Elite ABC kit, Vectorlab). Hematoxylin was used for counterstaining, and the images were visualized and captured using the Axioplan Imaging 2e microscope (Carl Zeiss, Oberkochen, Germany).

### 2.9. Xenograft Mouse Experiments

All laboratory mice were housed on a 12 h light/dark cycle in an animal facility at the University of Cincinnati. All experiments on mice in our research protocol were approved by the Institutional Animal Care and Use Committee (IACUC) at the University of Cincinnati. The 6–8-week-old BALB/c-nude female mice were purchased from Charles River. 1 × 10^7^ MCF7/TAM-Tet-on-miR-205 or BT474-Tet-on-miR-205 cells mixed with Matrigel (1:1) were injected into the fourth-mammary fat pads of the mice. Once tumors grew to the size of ~100 mm^3^, mice were randomly allocated to four groups with 4–6 mice/each and treated with a control vehicle, tamoxifen (Tam), doxycycline (Dox), or tamoxifen and doxycycline (Dox + Tam). The vehicle or 1 mg/mL Dox was supplemented in daily drinking water containing 5% sucrose. A total of 1 mg/mL of tamoxifen dissolved in sesame oil was subcutaneously injected into mice twice per week for seven weeks. Tumors were measured with Vernier calipers twice a week and the tumor volumes were calculated as volume (mm^3^) = π × length × width^2^/6. The impact of treatments on mice’s health, including appearance, behavior, and posture, such as hunching, hypoactivity, unkempt fur, and modulations in the elimination of wastes, etc., were monitored on a daily basis, and an animal under significant discomfort was immediately sacrificed to minimize pain and discomfort. Furthermore, when animals became moribund, or when the tumor reached 15% of the body weight, they were euthanized. At the end of the study, tumors were harvested, 10% formalin-fixed, and embedded in paraffin. Tumor sections were made and subjected to standard H&E and IHC staining using anti-MED1 and anti-Ki67 antibodies.

### 2.10. Patient Datasets and Statistical Analysis

NCBI GEO datasets (GSE19783 and GSE22220) were accessed for miRNA and mRNA expression data for human primary breast tumors. Correlations and statistical analyses were carried out with GraphPad Prism 5 (GraphPad Software Inc., La Jolla, CA, USA). The χ^2^-test was used to analyze the relationship between miRNA and target expression. Survival curves were plotted using the Kaplan–Meier method and compared by log-rank test. All data were analyzed using Sigmaplot (Version 12.3, Systat Software Inc., Germany) and GraphPad Prism software (version 9.0), and results were presented as mean ± standard deviation. Clinical data analysis was analyzed by SPSS software (version 20.0, IBM-SPSS Inc., Chicago, IL, USA). A comparison of two groups was performed by Student’s *t* test, while one-way analysis of variance (ANOVA) was performed for the comparison of multiple groups. A *p* value < 0.05 was considered statistically significant.

## 3. Results

### 3.1. miR-205 Inversely Correlated with MED1 Expression in Tamoxifen-Resistant Breast Cancers

**Figure 1:**
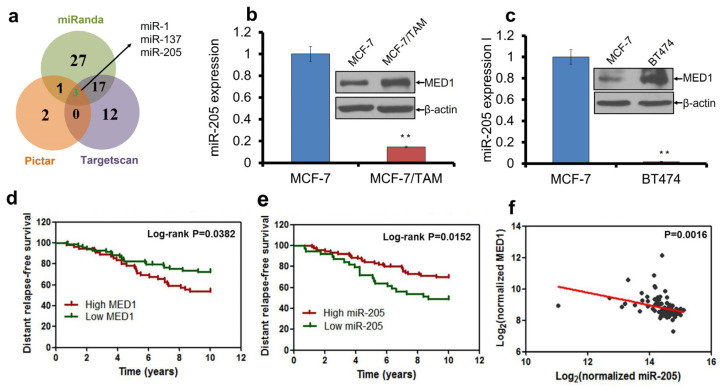
miR-205 expression inversely correlates with MED1 in tamoxifen-resistant breast cancer. (**a**) Venn diagram showing that miR-205 is predicted to target MED1 by three bioinformatics programs. (**b**,**c**) miR-205 expression and MED1 protein levels control MCF-7 and tamoxifen-resistant MCF-7/TAM (**b**) and BT474 (**c**) breast cancer cells. (**d**,**e**) Kaplan–Meier analysis of distant relapse-free survival curves for breast cancer patients with high or low expression of MED1 (**d**) or miR-205 (**e**) (n = 129). (**f**) Negative correlation between MED1 and miR-205-5p levels in human breast cancer by regression analysis. U6 small nuclear RNA was used as an endogenous control for miR-205 expression analysis and β-actin as sample-loading control in Western blot. The *p* value was determined by a log-rank test (** *p* ≤ 0.001). The data support MED1 as a miR-205 target and their inverse correlation in patient datasets, as well as potential roles in patient relapse-free survival and treatment resistance.

To investigate the molecular mechanisms controlling MED1 expression in breast cancer, we used three different microRNA target prediction programs (Pictar, TargetScan and miRanda) to screen for miRNAs that target MED1 [39,40]. We found three miRNAs were predicted by all three tools to target the MED1 3′UTR (miR-1, miR-137, and miR-205), with miR-205 having the highest score and multiple binding sites in MED1 3′-UTR (Figure 1a). Interestingly, we found that miR-205 was downregulated in both acquired tamoxifen-resistant cell line MCF-7/TAM (Figure 1b) and de novo tamoxifen-resistant cell line BT474 by quantitative RT-PCR analyses (Figure 1c). Importantly, both MCF-7/TAM and BT474 cells are also known to have a high expression of MED1 compared to control tamoxifen-sensitive MCF-7 cells (Figure 1b,c). To further examine the potential relevance of miR-205 and MED1 expression in human breast cancer patient samples, we analyzed a breast cancer patient dataset from the NCBI GEO database (GSE22220). The data indicated that patients with high MED1 expression levels (Figure 1d) or low miR-205 expression levels (Figure 1e) in tumors had much worse distant relapse-free survival than those with low MED1 expression levels or high miR-205 expression levels in Erα-positive breast cancer patients treated with tamoxifen therapy. Further analysis also showed a significant negative correlation between MED1 and miR-205 levels in these tumor samples (Figure 1f). Together, these data suggest the possible regulation of MED1 by miR-205 in mediating disease relapse and tamoxifen resistance in breast cancer.

### 3.2. miR-205 Regulates MED1 Expression by Targeting MED1 3′UTR

**Figure 2:**
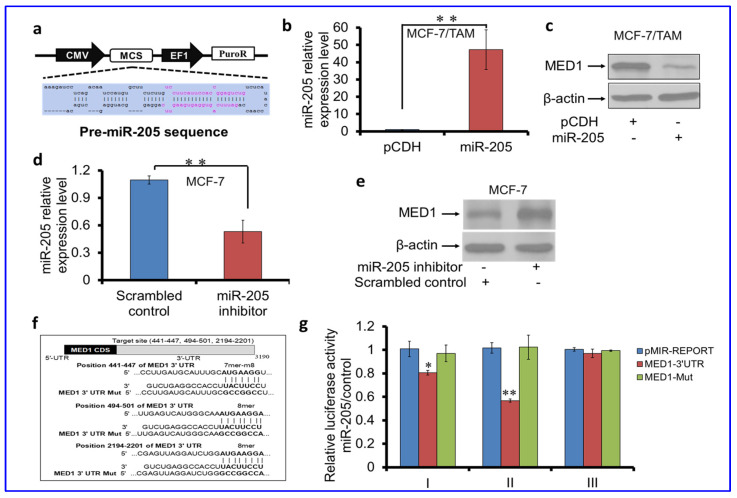
miR-205 targets and regulates MED1 expression in tamoxifen-resistant breast cancers. (**a**) miR-205 sequence and expression construct diagram. (**b**,**c**) qRT-PCR (**b**) and Western blot (**c**) analysis of miR-205 expression and MED1 protein levels in MCF-7/TAM cells transfected with vector control (pCDH) or miR-205 expression plasmids. (**d,e**) miR-205 expression (**d**) and MED1 protein level (**e**) in MCF-7 cells transfected with scrambled control or miR-205 inhibitors. (**f**) A schematic displaying miR-205 target and mutant sequences in the 3′UTR of MED1. (**g**) Luciferase assays of MCF-7 cells transfected with the pMIR-REPORT, pMIR-MED1-3′UTR, or pMIR-MED1-Mut with miR-205. U6 small nuclear RNA was used as an endogenous control for miR-205 expression analysis and β-actin as sample-loading control in Western blot. The data indicates that miR-205 specifically targets MED1 3′-UTR and ectopic MED1 expression, attenuating MED1 expression in MCF7/TAM cells but enhancing MED1 expression in MCF7 cells. Error bars represent the mean ± s.d. of three independent experiments. * *p* < 0.05, ** *p* < 0.01.

To further examine the regulation of MED1 by miR-205, we first transfected cells with miR-205 expression plasmid and found it can significantly decrease the MED1 protein levels in tamoxifen-resistant MCF-7/TAM (Figure 2a–c) and BT474 (Supplementary Figure S1) cells. Conversely, we used a miR-205 inhibitor in MCF-7 cells that have a higher level of miR-205 and found it can up-regulate the MED1 protein levels in these cells (Figure 2d,e). Furthermore, we examined whether miR-205 regulates MED1 expression via its 3′UTR through luciferase reporter assays. We generated three luciferase reporter constructs by inserting MED1 3′-UTR containing each of the three potential target sites (І 441-447, II 494-501, and III 2194-2201) of miR-205 (Figure 2f), respectively, along with mutations in the seed regions to prevent miR-205 targeting. These plasmids were transfected along with the CMV-pRL plasmid to control for variations in transfection efficiency, etc. The results indicated that luciferase activity was significantly suppressed by miR-205 when transfected with the reporter plasmids containing wild-type MED1 3′-UTR of site І or II, whereas MED1 3′-UTR in site III reporter gene activity was unchanged (Figure 2g). Importantly, the mutation of these predicted target sites relieved the suppressive effect of miR-205 expression (Figure 2g). Taken together, these results support that miR-205 can directly regulate the expression of MED1 through its 3′-UTR in breast cancer cells.

### 3.3. miR-205 Attenuated Tamoxifen Resistance Through Targeting MED1

**Figure 3:**
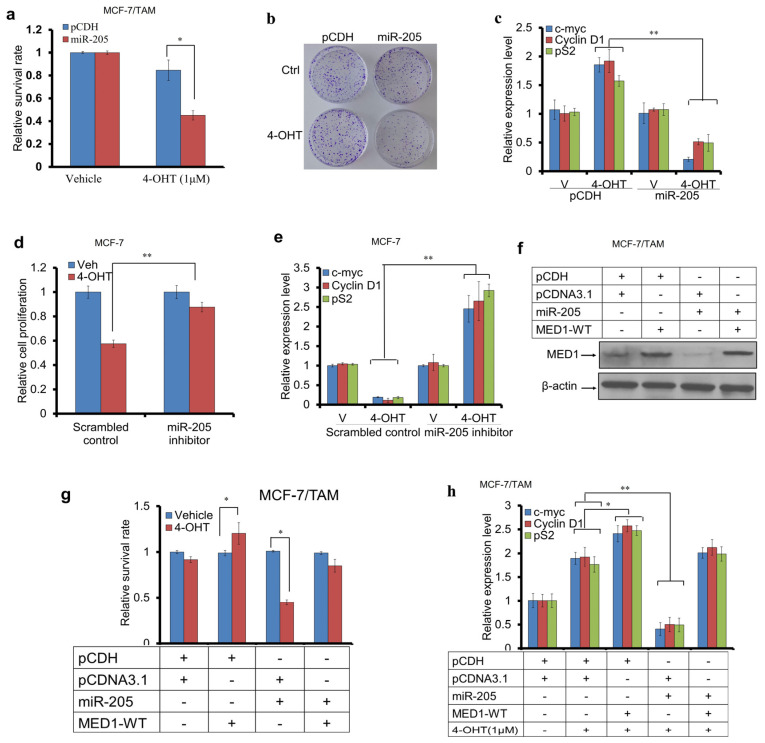
The overexpression of miR-205 sensitizes tamoxifen-resistant cells to tamoxifen treatment. (**a**,**b**) Cell proliferation (**a**) and colony formation assays (**b**) of MCF-7/TAM cells transfected with control vector (pCDH) or miR-205 expression plasmids and treated with vehicle or 4-OHT. (**c**) qRT-PCR analysis of c-myc, Cyclin D1, and pS2 expression in MCF-7/TAM cells treated as above. (**d**) Cell proliferation assay of MCF-7 cells transfected with scramble control, or miR-205 inhibitors as treated with vehicle or 4-OHT. (**e**) qRT-PCR analysis of c-myc, Cyclin D1, and pS2 expression in MCF-7 cells treated as in (**d**). (**f**) Western blot analysis of MED1 protein levels in MCF-7/TAM cells transfected with control or plasmids expressing miR-205 or MED1-WT as indicated. (**g**) Cell proliferation assay of MCF-7/TAM cells transfected with control or plasmids expressing miR-205, MED1-WT and treated with vehicle or 4-OHT. (**h**) qRT-PCR analysis of c-myc, Cyclin D1, and pS2 expression in MCF-7/TAM cells treated as in (**g**). GAPDH was used as control for the detection of the gene mRNA expression level and β-actin was used as the sample-loading control in Western blot. Error bars represent the mean ± s.d. of three independent experiments. * *p* < 0.05, ** *p* < 0.01. The data indicates that ectopic miR-205 expression enhances tamoxifen sensitivity in MCF7/TAM cells while inhibition of miR205 expression in MCF7 cells confers tamoxifen resistance based on cell growth, colony formation, and gene expression analyses.

Since miR-205 expression was low in tamoxifen-resistant cell lines, we hypothesized that the restoration of miR-205 expression would re-sensitize these cells to tamoxifen treatment. Indeed, the ectopic overexpression of miR-205 in MCF-7/TAM (Figure 3a) and BT474 (Supplementary Figure S2a) cells re-sensitized them to tamoxifen treatment but had no effect on control MCF-7 cells using MTT assays (Supplementary Figure S2b). Further, clonogenic analyses also indicated that the ectopic expression of miR-205 significantly reduced the clonogenic ability of MCF-7/TAM cells in the presence of 4-OHT (Figure 3b). Moreover, we found that the ectopic expression of miR-205 could inhibit tamoxifen-induced expression of ERα target genes (pS2, cyclin D1 and c-myc) in MCF-7/TAM cells compared with control (Figure 3c). Similar results were obtained in BT474 cells (Supplementary Figure S2c) but not in control tamoxifen-sensitive MCF7 cells (Supplementary Figure S2d). To further investigate whether the loss of miR-205 may confer tamoxifen resistance, we treated tamoxifen-sensitive MCF7 cells with the above miR-205 inhibitor. As shown in Figure 3d, the cells treated with miR-205 inhibitor became resistant to the treatment of 4-OHT with an upregulated MED1 protein expression (see Figure 2b, right panel). Moreover, the inhibition of miR-205 also enhanced the tamoxifen-induced expression of ERα target genes in MCF-7 cells (Figure 3e). Finally, to examine whether MED1 is essential for the above phenotypes, we created a miR-205 resistant MED1 construct which lacks the 3′ UTR. Western blot results confirmed that the ectopic expression of this MED1 construct (MED1-WT, without 3′UTR) could restore MED1 expression in MCF-7/TAM cells despite miR-205 overexpression (Figure 3f). Cell proliferation assays demonstrated that the ectopic expression of MED1 could rescue the tamoxifen sensitivity induced by miR-205 in MCF-7/TAM cells (Figure 3g). Additionally, the re-expression of MED1 rescued ERα target mRNA levels reduced by miR-205 in the presence of 4-OHT treatment (Figure 3h). Together, the data support the significance of miR205/MED1 axis in regulating the tamoxifen resistance of human breast cancer cells.

### 3.4. miR-205 Regulates MED1 Phosphorylation and Activation by Targeting HER3

**Figure 4:**
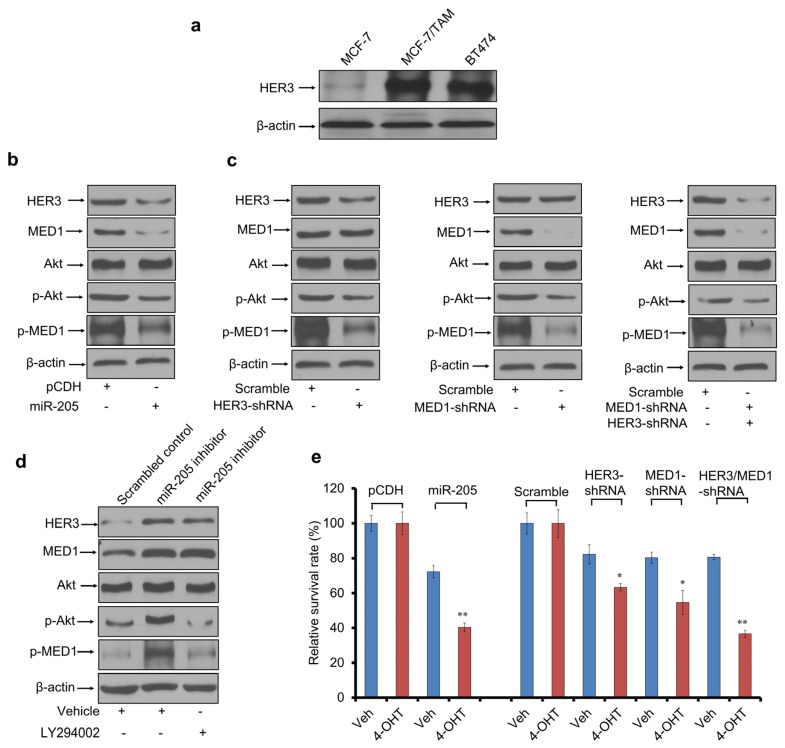
miR-205 regulates MED1 phosphorylation and activation through targeting HER3. (**a**) Western blot analysis of the expression of HER3 in tamoxifen-sensitive (MCF7) and tamoxifen-resistant (MCF-7/TAM and BT474) cells. (**b**,**c**) Western blot analyses of HER3, MED1, p-MED1, Akt and p-Akt in MCF-7/TAM cells transfected with control or miR-205-expressing plasmids (**b**), and in combination with HER3 shRNA, MED1 shRNA, or both (**c**). β-actin served as the loading control. (**d**) Western blot analysis of the expression of HER3, MED1, p-MED1, Akt and p-Akt in MCF-7 cells transfected with miR-205 inhibitor, LY294002, or both. (**e**) Relative cell survival of MCF-7/TAM cells transfected with control or miR-205 expressing plasmids with HER3 shRNA, MED1 shRNA, or both as indicated. β-actin was used as sample-loading control in Western blot. Error bars represent the mean ± s.d. of three independent experiments. * *p* < 0.05, ** *p* < 0.01. The data support the regulation of MED1 expression and phosphorylation by miR-205/HER3-mediated Akt signaling in the tamoxifen resistance of MCF7/TAM and BT474 cells.

We have previously established MED1 as a key crosstalk point for HER2 and ERα pathways in mediating tamoxifen resistance by increasing MED1 phosphorylation [38]. Interestingly, the HER2 dimerization partner HER3 has been reported to be a target of miR205 in breast cancer cells [41]. Indeed, we found that HER3 is also overexpressed in tamoxifen-resistant MCF-7/TAM and BT474 cells compared to that of MCF-7 cells. (Figure 4a). In silico analysis of HER3 3′-UTR indicated a highly conserved miR-205 binding site. Further, by using the HER3 3′-UTR luciferase reporter assay, we found that the overexpression of miR-205 decreased while the inhibition of miR-205 increased its luciferase activities (Supplementary Figure S3a). On the contrary, this regulation is abrogated by mutations in the HER3 3′-UTR. Moreover, consistent with this, we found that the expression level of HER3 was also reduced in miR-205-transduced MCF-7/TAM cells (Figure 4b). To establish the relevance of miR-205 and HER3 with human breast cancers, we analyzed a publicly available microarray dataset from the NCBI GEO database (GSE22220). Regression analysis showed a negative correlation between the normalized HER3 protein level and the normalized miR-205 level in human breast cancer (Supplementary Figure S3b). This analysis also revealed that patients with high HER3 expression levels in their tumors had much worse distant relapse-free survival than those with low HER3 expression levels (Supplementary Figure S3c).

Next, we examined whether the HER3/Akt pathway can also regulate MED1 phosphorylation in tamoxifen-resistant cells. First, we found that the expression of both phospho-AKT and phosphor-MED1 decreased following miR-205 overexpression (Figure 4b). Next, we carried out shRNA knockdown experiments using lentivirus-expressing HER3 shRNA and MED1 shRNA, alone or in combination. We found that HER3 knockdown was able to strongly reduce phospho-AKT and phospho-MED1 levels without affecting total levels of either protein, while the simultaneous knockdown of HER3 and MED1 achieves a comparable reduced MED1 expression and phosphorylation to what we observed with miR-205 overexpression (Figure 4c). To further test the role of AKT in MED1 phosphorylation, MCF-7/TAM cells were transfected with miR-205 inhibitor and treated with PI3K inhibitor LY294002. Western blot results showed that the upregulation of p-MED1 by miR-205 inhibitor in MCF-7 cells was strongly inhibited by LY294002 (Figure 4d). Finally, we carried out cell proliferation assays and found that the growth inhibition by miR-205 was similar to that of the simultaneous knockdown of MED1 and HER3 in the presence of 4-OHT (Figure 4e). All together, these data suggest that miR-205 regulates MED1 activity through both the direct repression of its expression and the reduction in its phosphorylation through HER3/AKT pathway.

### 3.5. miR-205 Regulates the Recruitment of MED1, p-MED1, N-CoR and SMRT to ER Target Gene Promoter

**Figure 5:**
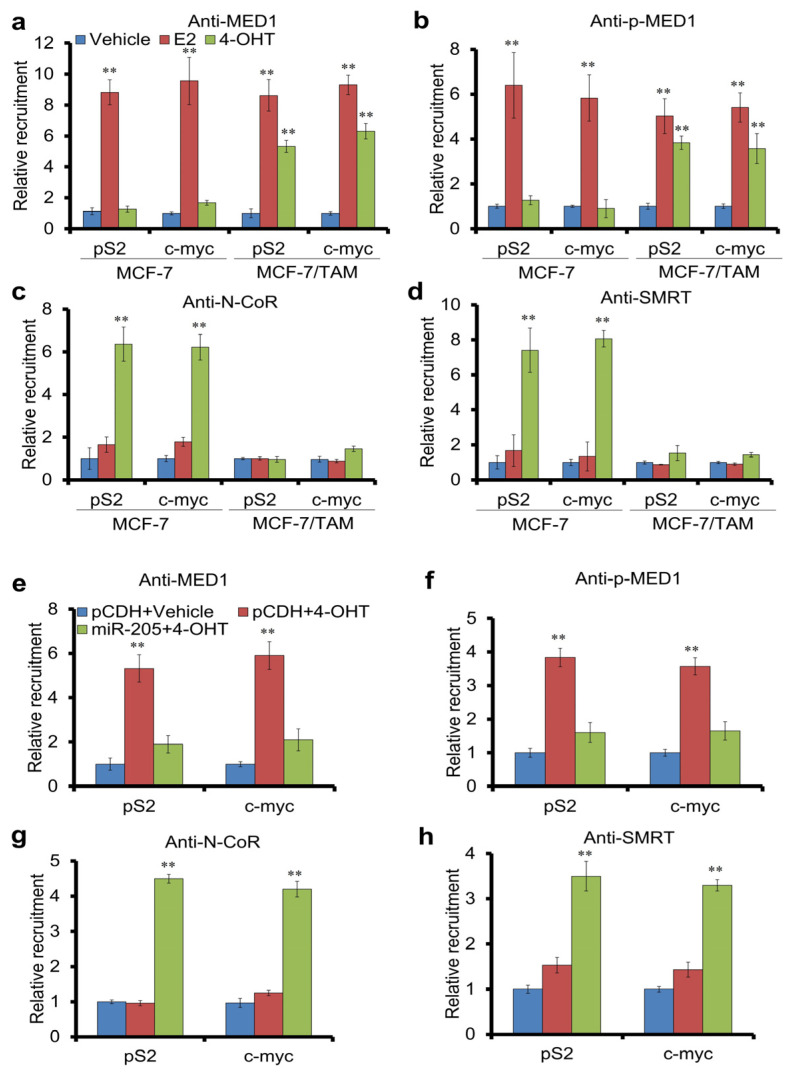
miR-205 regulates the recruitment of MED1, p-MED1, N-CoR and SMRT to ERα target gene promoter by tamoxifen. (**a**–**d**) The results of chromatin immunoprecipitation in MCF-7 and MCF-7/TAM cells were treated with 100 nmol/L E2 or 1 mmol/L 4-OHT for 45 min and subjected to chromatin immunoprecipitation experiments using control IgG or antibodies against MED1 (**a**), p-MED1 (**b**), N-CoR (**c**), or SMRT (**d**). Immunoprecipitated DNA corresponding to the pS2 or c-myc promoter region was amplified and quantified by qRT-PCR. (**e**–**h**) The results of chromatin immunoprecipitation experiments carried out using control IgG or antibodies against MED1 (**e**), p-MED1 (**f**), N-CoR (**g**), or SMRT (**h**) as above in MCF-7/TAM cells transfected with control or miR-205-expressing plasmids in the presence of vehicle or 4-OHT. Error bars represent the mean ± s.d. of three independent experiments. ** *p* < 0.01. Our data indicate that the expression of miR-205 restores the tamoxifen-induced recruitment of the ER co-repressors N-CoR and SMRT, and not the co-activator MED1 to the ER target genes pS2 and c-Myc promoters in tamoxifen-resistant cells.

N-CoR and SMRT are transcriptional co-repressors recruited by ER to target gene promoters upon tamoxifen treatment to repress gene expression and inhibit breast cancer cell growth [40]. We have previously found that activated MED1 displaces these corepressors on ER target genes’ promoters to promote transcription. [36]. To further explore the mechanism of miR-205 in affecting the tamoxifen resistance of breast cancer cells, we conducted ChIP assays using parent MCF-7 and treatment-resistant MCF-7/TAM cells. We found that tamoxifen treatment inhibited the recruitment of MED1 and p-MED1 to ER target genes in parental cells compared to that of estrogen, as expected. However, tamoxifen treatment was found to induce their recruitment to the target genes’ promoters in MCF7/TAM cells instead (Figure 5a,b). Conversely, while N-CoR and SMRT were not recruited to these sites in either cell line under the vehicle or estrogen treatment, tamoxifen treatment resulted in their recruitment in parental MCF-7 but not MCF7/TAM cells (Figure 5c,d). To examine the role of miR-205 in the promoter recruitment of these cofactors, we repeated the ChIP assays using MCF7/TAM cells transformed with control or miR-205 expressing plasmids. Significantly, we found that the expression of miR-205 is able to decrease the MED1 and p-MED1 recruitment (Figure 5e,f) and restore the tamoxifen-induced recruitment of ER-corepressors (Figure 5e–h). Taken together, our data support that miR-205 regulates the breast cancer response to tamoxifen and target gene expression by affecting the recruitment of transcriptional coactivators and corepressors to the target gene promoter.

### 3.6. Overexpression of miR-205 Re-Sensitizes Tamoxifen-Resistant Breast Cancer Cells to Tamoxifen Treatment In Vivo

**Figure 6:**
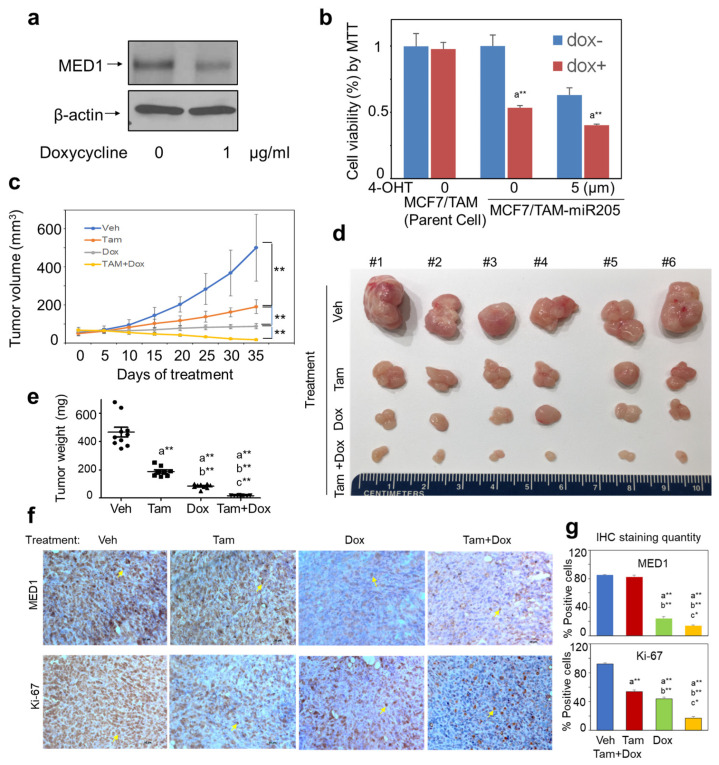
The overexpression of miR-205 re-sensitizes tamoxifen-resistant breast cancer cells to tamoxifen in vivo. MCF-7/TAM-tet-miR-205 cells mixed with Matrigel (1:1) were injected into the fourth-mammary fat pads of the mice and randomly allocated to four groups for Dox and Tamoxifen treatment as indicated, once tumors reached the size ~100 mm^3^. (**a**) MED1 expression was decreased by Dox in MCF-7/TAM-tet-miR-205 cells. (**b**) MCF-7/TAM-tet-miR-205 cell proliferation in the presence of Dox and/or 4-OHT. (**c**) Growth curves of xenograft tumors (n = 4–6/group). (**d**) Images of excised tumor tissues from control and treatment groups, with a ruler for scale. (**e**) Tumor weights of excised tumor tissues from the control and treatment groups. (**f**) IHC staining of excised tumor tissues showing the expression changes in MED1 (top panel) and Ki67(bottom panel) in tumors of the control and treatment groups. (**g**) The quantitative results of IHC staining of MED1 (top panel) and Ki67 (bottom panel) expression in tumors of excised tumor tissues from the control and treatment groups. β-actin was used as sample-loading control in Western blot. * *p* < 0.05, ** *p* < 0.01. a: vs. Veh, b: vs. Tam, c: vs. Dox. The data indicate that induced miR-205 expression in MCF7/TAM cells inhibits MED1 expression and tumor growth and enhances tamoxifen treatment sensitivity in vivo in the orthotopic xenograft mouse model.

We have demonstrated the role of miR-205 in regulating the tamoxifen resistance of breast cancer cells through MED1 in vitro in our above studies. To further examine the effect of miR-205 overexpression in tamoxifen-resistant breast cancer cells on tamoxifen sensitivity in vivo, we established MCF-7/TAM and BT474 sublines that stably express tet-inducible miR-205 (MCF-7/TAM-tet-miR-205 and BT474-tet-miR-205). As expected, the addition of doxycycline significantly downregulated the MED1 protein levels, reduced cell proliferation, and sensitized these cells to tamoxifen treatment (Figure 6a,b and Supplementary Figure S4a,b). Next, we orthotopically implanted MCF-7/TAM-tet-miR-205 and BT474-tet-miR-205 cells mixed with Matrigel orthotopically into the mammary fat pads of nude mice. Once the tumors reached a size of about 50 mm^3^, mice were randomized into four treatment groups: vehicle control (Veh), tamoxifen alone (Tam), doxycycline alone (Dox), and combination (Tam + Dox). Tumor volume was measured, and the growth curves were plotted accordingly, as shown in Figure 6c and Supplementary Figure S4c. We found that either tamoxifen or Dox treatment alone significantly reduced tumor growth compared with the vehicle treatment. Importantly, a combination of tamoxifen and doxycycline treatment further exhibited a greater inhibition of tumor growth in tumor size and weight (Figure 6d,e and Supplementary Figure S4d,e), supporting that miR-205 overexpression efficiently sensitized tumors to tamoxifen treatment. We carried out IHC staining and found that MED1 expression was dramatically decreased in tumor samples from treatment groups with Dox or a combination of Tam plus Dox (Figure 6f,g, and Supplementary Figure S4f,g, top panel). Further, the expression of the proliferation marker Ki67 was significantly reduced in tumor samples from all three treatment groups (Figure 6f,g, and Supplementary Figure S4f,g, bottom panel), with the lowest positive percentage and weakest staining strength of the Ki-67 marker in the samples from the combination treatment group (Tam plus Dox). Collectively, these in vivo results are indicative of the key roles of miR-205 in tamoxifen resistance and their potential use as a new therapeutic target and biomarker.

## 4. Discussion

Anti-estrogens, such as tamoxifen, have been widely used as the first-line adjuvant therapy for patients with Erα-positive breast cancer. However, most patients eventually develop resistance, which has become a major clinical obstacle limiting the success of breast cancer treatment [42,43]. We have previously reported that MED1, a key ER transcription coactivator, is overexpressed in about half of breast cancer and plays important roles in ERα-mediated transcription and tamoxifen resistance [13,14,16]. However, the molecular mechanism underlying this mechanism of treatment resistance is not very well understood. In this study, by using a combination of bioinformatics tools, de novo and acquired tam-resistant breast cancer cell lines, and human breast cancer patient datasets, we have identified miRNA-205 as a key regulator of MED1 expression and activation in mediating the endocrine resistance of human breast cancer, and further determined its roles and underlying molecular mechanisms in both in vitro and in vivo orthotopic xenograft mouse models.

MiR-205 is located on chromosome 1q32.2 and has been reported to regulate the growth/development of the breast, thymus, and prostate [44]. Interestingly, the differential expression of miR-205 is specific to tumor types with high expression in ovarian cancer, bladder cancer, and non-small cell lung cancer, and low expression in breast cancer, melanoma, renal cell cancer, and prostate cancer [45,46,47,48,49]. miR-205 has also been reported as either a tumor suppressor by inhibiting proliferation and invasion, or as an oncogene by facilitating tumor initiation and proliferation, depending on the specific tumor context and target genes [50,51,52]. Among these, miR-205 has been mainly described as a potential tumor suppressor in breast cancer, and miR-205 expression has been reported to be epigenetically regulated by promoter DNA methylation [53]. Mouillet and colleagues showed MED1 as potential target for MIR-205, as specific 3′UTR targeting attenuates/silences MED1 expression. One recent study also reported that the hypermethylation of the miR-205 locus was strongly correlated with decreased miR-205 expression and increased MED1 expression in primary prostate cancer samples, hypoxic primary human trophoblasts, and obesity/hyperleptinemia-induced tamoxifen resistance [54,55,56]. However, the role and molecular mechanisms of miR-205 and its regulation of MED1 in breast cancer and its treatment resistance is not very well understood. In this study, we reported that miR-205 plays an important role in both de novo and acquired tamoxifen resistance in ERα-positive breast cancer in vitro and in vivo orthotopic xenograft mouse models. Restoring miR-205 expression could re-sensitize the tamoxifen treatment response and might represent a viable strategy to combat tamoxifen resistance in human breast cancer. Significantly, our experimental results are supported by clinical data from publicly available databases, where we found a statistically significant negative correlation between the expression of miR-205 and MED1 in tamoxifen-treated patients. Importantly, the lower expression of miR-205 was correlated with poorer disease-free survival in tamoxifen-treated patients. Importantly, the lower expression of miR-205 was correlated with poorer disease-free survival in tamoxifen-treated patients. It will be important in the future to further validate these findings using human patient-derived samples, and eventually in clinical trials for its use as a biomarker and/or therapeutic target, in order to have an impact on patients.

We have previously reported that HER2 signaling can activate MED1 phosphorylation via the MAPK signaling pathway to mediate tamoxifen resistance [38]. HER2 could be activated by dimerization with another HER family receptor. Of these, the HER2/HER3 heterodimer is the most potent oncogenic complex. Importantly, HER3 has also been reported to be frequently overexpressed in breast cancer, and the activation of HER3 signaling was recently also reported to contribute to the resistance to HER2-targeted therapy [57,58,59,60]. Interestingly, we found that miR-205 can not only regulate MED1 expression but also the expression of HER3 through its 3′ UTR. HER3 has been reported to bind the p85 regulatory subunit of PI3K and activate the PI3K/Akt signaling pathway. Further, the activation of PI3K/Akt signaling has also been reported to be associated with breast cancer tamoxifen resistance [61,62,63]. Consistent with these notions, we found that the inhibition of HER3 or PI3K/Akt can significantly reduce the MED1 phosphorylation, supporting a dual role for miRNA-205 in regulating MED1 expression through its 3′UTR and MED1 phosphorylation/activation by regulating the HER3/PI3K/Akt signaling. Mechanistically, we found that this resulted in the promoters of ERα-target genes being occupied by transcriptional coactivator MED1 in tamoxifen-resistant breast cancer cells rather than the transcriptional corepressors N-CoR and SMRT. Thus, the ectopic expression of miR-205 is able to reduce MED1, HER3, and HER3-induced MED1 phosphorylation, thereby re-sensitizing cells to tamoxifen treatment with restored transcriptional corepressor recruitment. Although our data support the direct regulation of MED1 and HER3 by miR-205, we cannot exclude that there are other factors that may also directly or indirectly contribute to their regulation.

Together with our previous findings, HER2-mediated MED1 activation through MAPK signaling and our current findings on the miR-205 regulation of both HER3/PI3k/Akt and MED1 suggest a complex molecular interaction and crosstalk between the HER2, HER3, PI3K-AKT, MAPK signaling axes, where MED1 and miR-205 could play crucial roles in regulating the treatment resistance of breast cancer. Importantly, the PI3K/AKT pathway has been recognized to play key roles in anti-estrogen resistance of human breast cancer, with multiple inhibitors already developed and used in the clinics for patients who have failed earlier lines of endocrine therapy [64]. However, these inhibitors often show limited efficacy with high toxicity and serious adverse effects, and patients quickly relapse [65]. It will be interesting to further examine whether targeting the miR-205/MED1 axis alone or in combination with these current therapies could provide a more advantageous impact to overcome treatment resistance with better efficacy and improved side-effect profiles. Moreover, there are a number of intrinsic and extrinsic factors have also been reported to be involved in anti-estrogen treatment resistance of human breast cancer, it will be important to further study the biological impact of miR-205/MED1 axis, both within tumor cells including tumor stem cells, and in the interaction and communication of tumor cells with the tumor microenvironment (e.g., exosomes, adipose cells, MSCs, immune cells) in the future [66,67,68,69].

## 5. Conclusions

Our study supports that miR-205 plays an important role in mediating tamoxifen resistance through regulating both MED1 expression and activation, and, thus, miR-205 re-expression could represent a more efficient and advantageous approach to overcome tamoxifen resistance in breast cancer. Importantly, our lab has recently established an RNA nanotechnology-based approach to specifically target breast cancer cells for the delivery of small RNAs and achieved highly desirable efficacy with no apparent toxicity. We therefore could further adapt this system to restore the miR-205 expression as a potential future treatment strategy. Since our lab has previously demonstrated the role of MED1 in the response to another anti-estrogen fulvestrant, and our recent publication indicated a potential role of MED1 in the treatment resistance to anti-HER2 therapy, miR-205-based therapy could therefore be applied in these settings, and possibly in others as well. It is of critical importance to test these out further in the future in more in vitro and in vivo human patient-derived preclinical tumor models and, most importantly, in the clinics, to examine their potential utilities for the benefit of breast cancer patient care.

## Data Availability

The data generated in this study are available within the article and its supplementary data files.

## Supplementary Materials

The following supporting information can be downloaded at: https://www.mdpi.com/article/10.3390/cancers16233992/s1, Figure S1: miR-205 regulates MED1 protein expression level in breast cancer; Figure S2: miR-205 attenuated tamoxifen resistance; Figure S3: miR-205 targets and relevance with HER3; Figure S4: Overexpression of miR-205 re-sensitizes tamoxifen-resistant breast cancer cells to tamoxifen *in vivo*. Table S1: Primer list and sequences.
