# Supplementary material for: miR-205 Regulates Tamoxifen Resistance by Targeting Estrogen Receptor Coactivator MED1 in Human Breast Cancer
Source: Cancers (Basel). 2024 Nov 28;16(23):3992. doi: 10.3390/cancers16233992 (PMC11640040; doi:10.3390/cancers16233992)

Supplementary Figures:

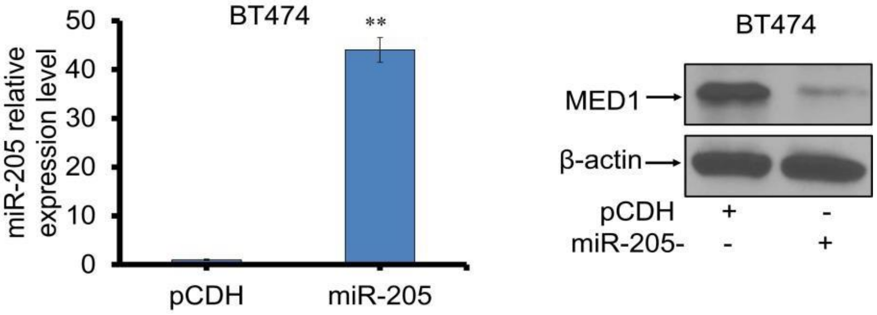

Figure S1. miR-205 regulates MED1 protein expression level in breast cancer. BT474 cells transfected with miR-205-pCDH (miR-205) expressing or control (pCDH) vector. miR-205 expression was determined by qPCR (left panel) and MED1 protein level was determined by western blot. Error bars represent the mean ± s.d. of three independent experiments. ** $p < 0.01$.

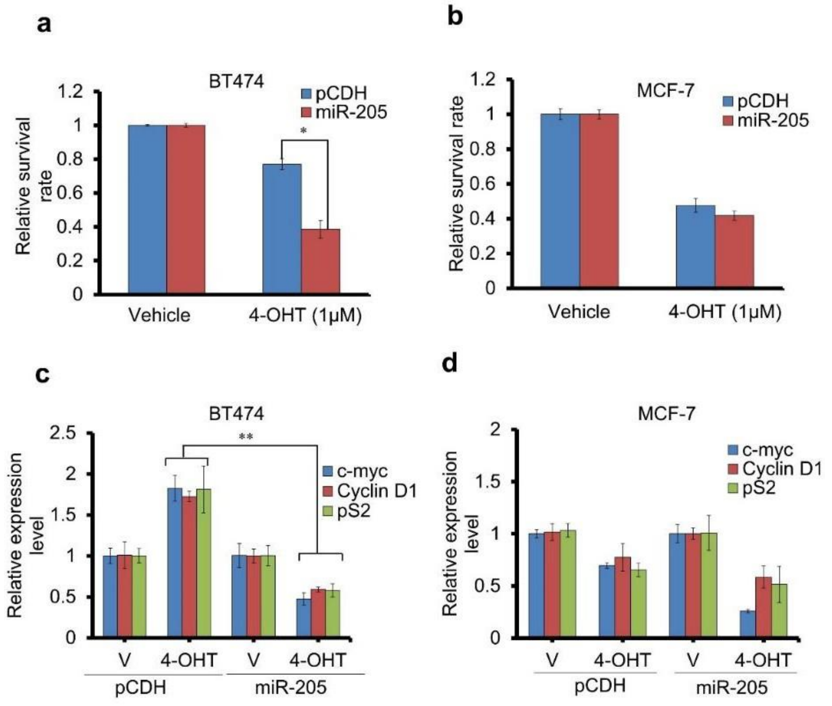

Figure S2. miR-205 attenuated tamoxifen resistance. BT474 and MCF-7 cells were transfected with miR- 205-pCDH (miR-205) expressing or control (pCDH) vector and treated with control or 1 μM 4-OHT. Cell proliferation by MTT assay and transcription quantification of ERα-target genes (c-myc, Cyclin D1, and pS2) by qPCR were followed.

(a) overexpression of miR-205 increased the sensitivity of BT474 to tamoxifen treatment **(b)** overexpression of miR-205 had no impact on the sensitivity of MCF-7 to tamoxifen treatment. **(c and d)** overexpression of miR-205 inhibited transcriptions of ERα-target genes in the presence of 4-OHT in BT474 but not significantly in MCF7 cells. Error bars represent the mean ± s.d. of three independent experiments. * $p < 0.05$, ** $p < 0.01$.

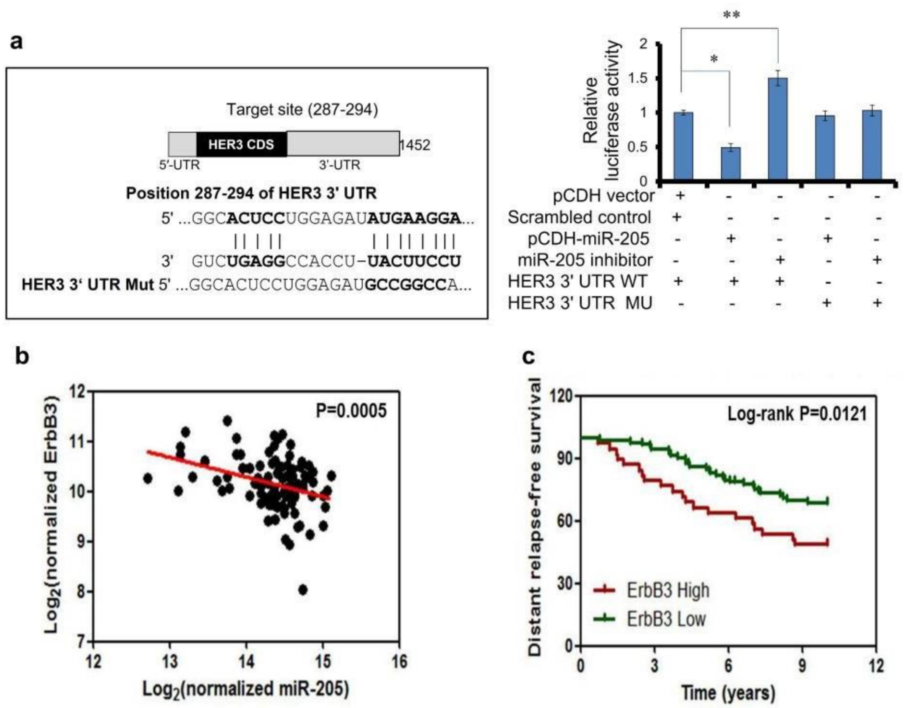

Figure S3. miR-205 targets and relevance with HER3. (a) Left panel: predicted miR-205 target sequence in HER3-3′UTR. Right panel: Luciferase assay of MCF-7 cells transfected with the pMIR-REPORT, pMIR-HER3-3'UTR or pMIR-HER3-Mut with pCDH-miR-205, miR-205 inhibitor or scrambled control. **(b)** Analysis of the publicly available microarray dataset from the NCBI GEO database (GSE22220) showed that a negative correlation between the normalized HER3 protein level and the normalized miR-205 level is revealed by Regression analysis, and **(c)** high HER3 expression levels in tumors had much worse distant relapse-free survival than those with low HER3 expression levels in the human breast cancer. Error bars represent the mean ± s.d. of three independent experiments. * $p < 0.05$, ** $p < 0.01$.

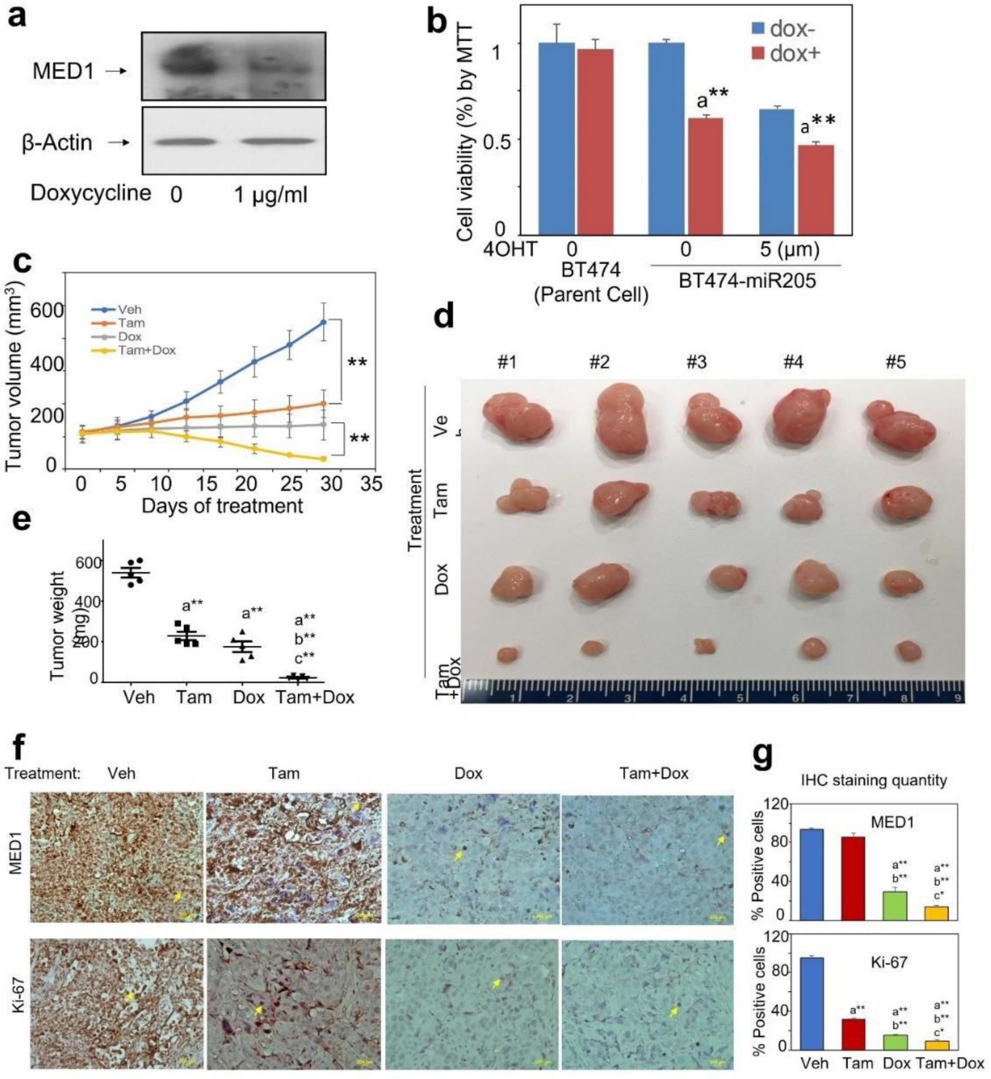

Figure S4. Overexpression of miR-205 re-sensitizes tamoxifen-resistant breast cancer cells to tamoxifen *in vivo*. (a) MED1 expression was decreased by doxycycline in BT474-tet-miR-205 cells. **(b)** Dox inhibited BT474-tet- miR-205 cell proliferation and enhanced the efficacy of 4-OHT. **(c)** Growth curves of xenograft tumors (n=8-10/group).

(d) The images of tumors from each group. **(e)** Tumor weights in each group (n=8-10/group). **(f)** IHC staining of MED1 (top panel) and Ki-67 (bottom panel) in tumors of each group. **(g)** Quantity of positive staining of MED1 (top panel) and Ki-67 (bottom panel) in tumor cells from each group. 1000 cells are examined from each group. Error bars represent the mean ± s.d. of three independent experiments. * $p < 0.05$, ** $p < 0.01$. a: vs. Veh, b: vs. Tam, c: vs. Dox.

Supplementary Table 1. Primer list and sequences

Name	used for	Sequence (5' - 3')
U6-F	qPCR	CTCGCTTCGGCAGCACA
U6-R	qPCR	AACGCTTCACGAATTTGCGT
miR205-RT	qPCR	GTCGTATCCAGTGCAGGGTCCGAGGTATTCGCACTGGA TACGACCAGACT
miR205-F	qPCR	GCCTGTCCTTCATTCCACCG
miR205-R	qPCR	GTGCAGGGTCCGAGGT
pS2-F	qPCR	CGTGAGCCACTGTTGTCAGG
pS2-R	qPCR	TGGTGAGGTCATCTTGGCTG
c-myc-F	qPCR	GAAGAAATTCGAGCTGCTGC
c-myc-R	qPCR	CACATACAGTCCTGGATGATG
Cyclin D1 -F	qPCR	TGGAGGTCTGCGAGGAACAGAA
Cyclin D1 -R	qPCR	TGCAGGCGGCTCTTTTTCA
GPADH-F	qPCR	CGGAGTCAACGGATTTGGTCGTA
GPADH -R	qPCR	AGCCTTCTCCATGGTGGTGAAGAC
MED1 -F	qPCR	AAGGCTCAGCCCAGACTTTATG
MED1 -R	qPCR	CCCCTTATGGTGGTTTGCCTA
HER3-3'UTR-F	luciferase assay	ATCACTAGTGTAACTCCTGCTCCCTGTGG
HER3-3'UTR-R	luciferase assay	ATAATACGCGTTGAATTTGCCCTCGGATAAG
HER3-3'UTR-Mutation-1-F	luciferase assay	AGGCACTCCTGGAGATGCCGGCCATTACTCTCCATATCC
HER3-3'UTR-Mutation-1-R	luciferase assay	GGATATGGAGAGTAATGGCCGGCATCTCCAGGAGTGCCT
MED1-3'UTR-F	luciferase assay	ATCACTAGTATAGGCAAACCACCATAAGG
MED1-3'UTR-R	luciferase assay	TATAAGCTTATGCTGGGATGCAGACTTTTG
MED1-3'UTR-Mutation-1 -F	luciferase assay	TTTGAGTCATGGGCAAGCCGGCCACTTTGGTCATTTTGGA
MED1-3'UTR-Mutation-1 -R	luciferase assay	TCCAAAATGACCAAAGTGGCCGGCTTGCCCATGACTCAAA
MED1-3'UTR-Mutation-2 -F	luciferase assay	CCTTGATGCATTTGCGCCGGCCTTGTTCAACTTTGTT
MED1-3'UTR-Mutation-2 -R	luciferase assay	AACAAAGTTGAACAAGGCCGGCGCAAATGCATCAAGG
MED1-3'UTR-Mutation-3 -F	luciferase assay	CCGAGTTAGGATCTGGGCCGGCCGTAAGCCCCTGAATTGT
MED1-3'UTR-Mutation-3 -R	luciferase assay	ACAATTCAGGGGCTTACGGCCGGCCCAGATCCTAACTCGG
MED1-3'UTR3190-F	MED 1 w/o 3'UTR	ATCACTAGTGAACCTTATTTCCTAAAAGAAACA
MED1-3'UTR3190-R	MED 1 w/o 3'UTR	ATAATACGCGTTTACTTTTTATCAAAGAAGT
MED1-3'UTR920-F	MED 1 w/o 3'UTR	ATCACTAGTATCAGCTCTCAACAACTCTG
MED1-3'UTR920-R	MED 1 w/o 3'UTR	ATAATACGCGTAGCCTGGTTTTGGCAATTAT

Chip primer of pS2 promoter- F	ChIP	GTTTGTGACCCAGGCATCTT
Chip primer of pS2 promoter- R	ChIP	CAGGGTCCTGTCATTGTGTG
Chip primer of c-Myc promoter -F	ChIP	GAGCAGCAGAGAAAGGGAGA
Chip primer of c-Myc promoter -R	ChIP	CAGCCGAGCACTCTAGCTCT

Figure 1b

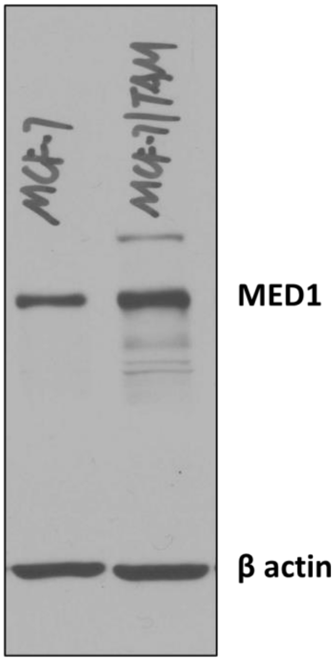

Figure 1c

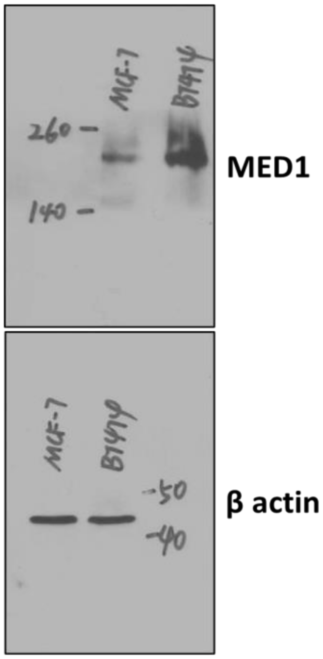

Figure 2a

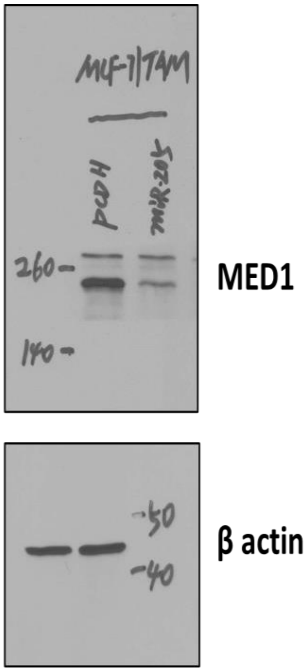

Figure 2b

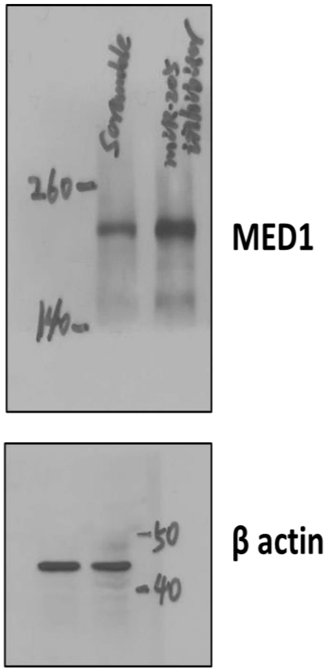

Figure 3f

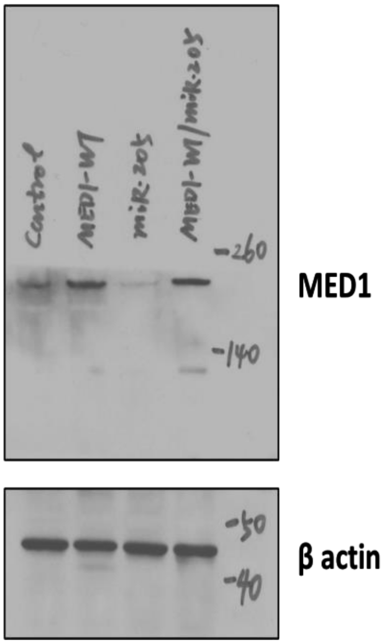

Figure 4a

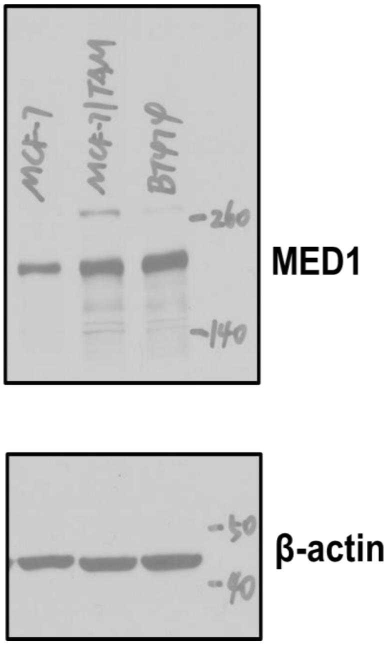

Figure 4 b-c

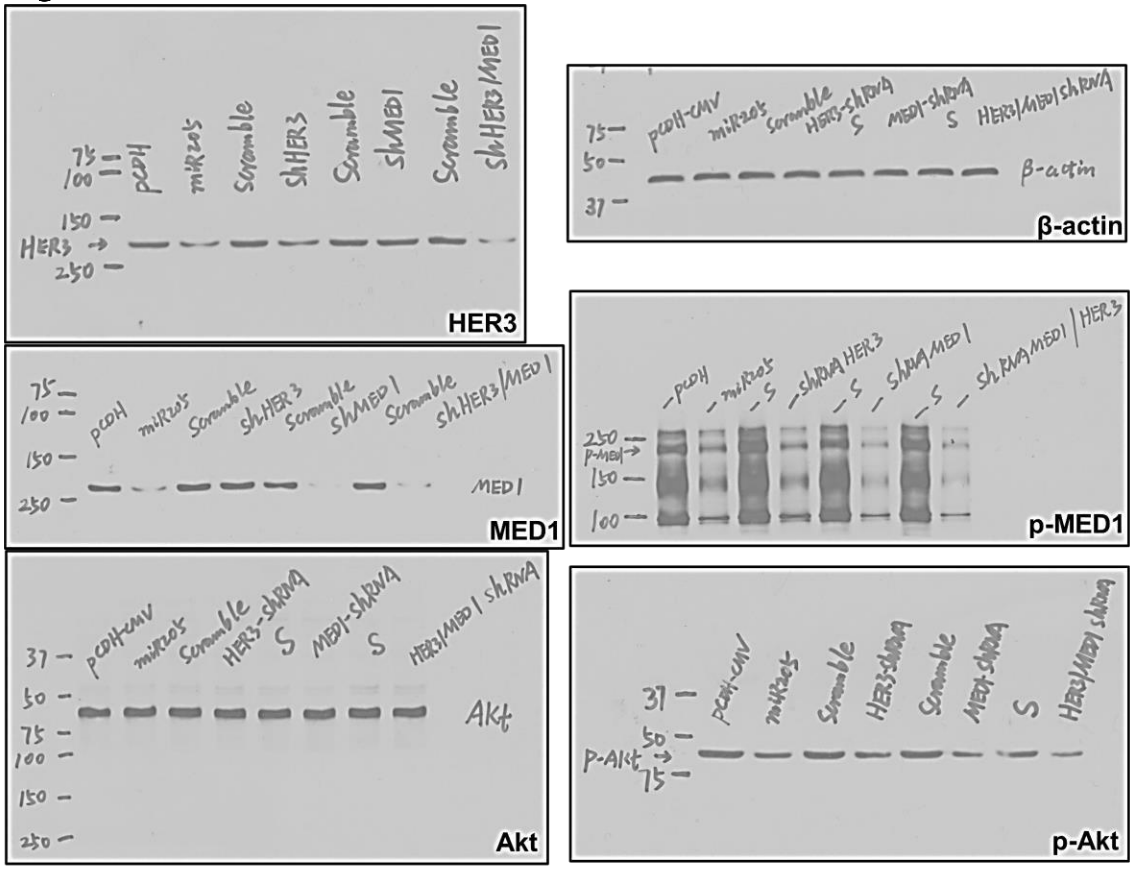

Figure 4d

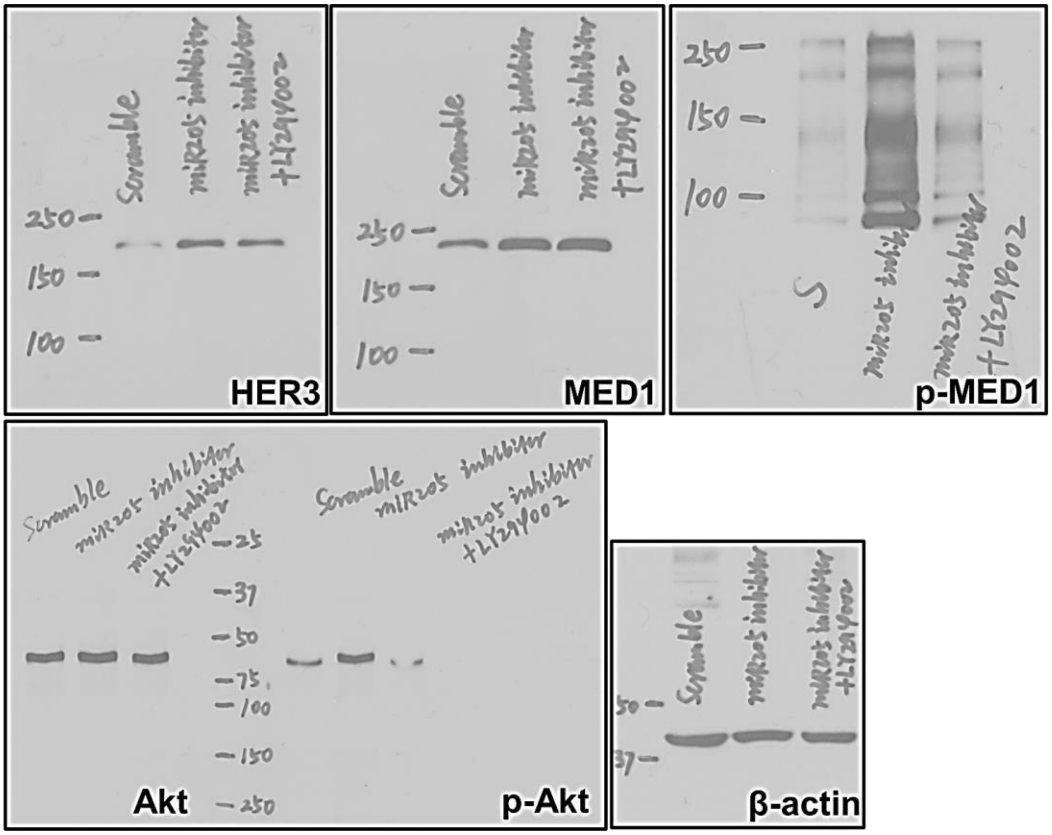

Figure 6a

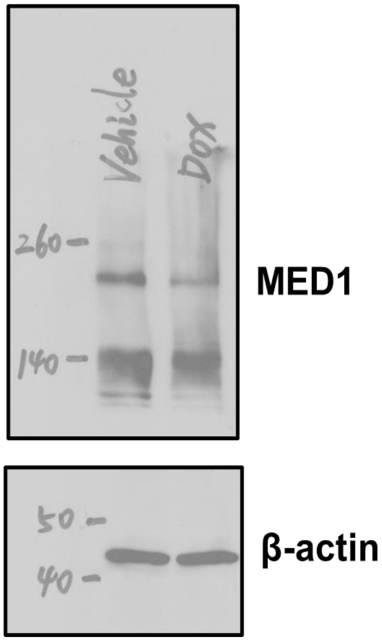

Supplemental Figure S1

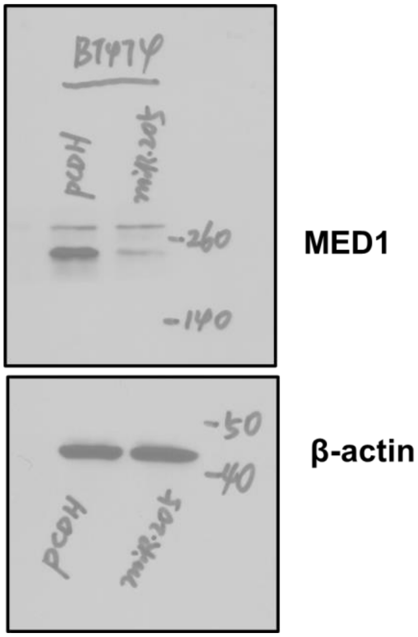

Supplement: Supplementary file 1 [file cancers-16-03992-s001.zip › cancers-3288310-supplementary.pdf]
